# Prediction of disease-associated mutations in the transmembrane regions of proteins with known 3D structure

**DOI:** 10.1371/journal.pone.0219452

**Published:** 2019-07-10

**Authors:** Petr Popov, Ilya Bizin, Michael Gromiha, Kulandaisamy A, Dmitrij Frishman

**Affiliations:** 1 Skolkovo Institute of Science and Technology, Moscow, Russia; 2 Moscow Institute of Physics and Technology, Dolgoprudny, Russia; 3 St Petersburg State Polytechnic University, St Petersburg, Russia; 4 Department of Biotechnology, Bhupat and Jyoti Mehta School of Biosciences, Indian Institute of Technology Madras, Chennai, Tamil Nadu, India; 5 Department of Bioinformatics, Wissenschaftzentrum Weihenstephan, Technische Universität München, Freising, Germany; Cincinnati Children's Hospital Medical Center, UNITED STATES

## Abstract

Being able to assess the phenotypic effects of mutations is a much required capability in precision medicine. However, most of the currently available structure-based methods actually predict stability changes caused by mutations rather than their pathogenic potential. There are also no dedicated methods to predict damaging mutations specifically in transmembrane proteins. In this study we developed and applied a machine-learning approach to discriminate between disease-associated and benign point mutations in the transmembrane regions of proteins with known 3D structure. The method, called BorodaTM (BOosted RegressiOn trees for Disease-Associated mutations in TransMembrane proteins), was trained on sequence-, structure-, and energy-derived descriptors. When compared with the state-of-the-art methods, BorodaTM is superior in classifying point mutations in transmembrane regions. Using BorodaTM we have conducted a large-scale mutation analysis in the transmembrane regions of human proteins with known 3D structures. For each protein we generated structural models for all point mutations by replacing each residue to 19 possible residue types. We classified ~1.8 millions point mutations as benign or diseased-associated and made all predictions available as a Web-server at https://www.iitm.ac.in/bioinfo/MutHTP/boroda.php.

## Introduction

Due to the advent of the next generation sequencing technologies and their quick foray into the clinical practice, predicting the phenotypic consequences of missense mutations has arguably become the most active and demanded area of applied bioinformatics. This is evidenced by the astronomic citation counts of the leading computational methods for this purpose, such as PolyPhen [1] and SIFT [2], and also by the fact that *in silico* tools have been officially made part of the standards and guidelines for the interpretation of sequence variants [3]. Virtually every medically oriented exome, genome, or transcriptome sequencing study is faced with the daunting task of selecting a small number of substitutions associated with a given phenotype from a vast amount of benign variants for subsequent experimental validation.

A large variety of algorithms for performing this task is currently available (reviewed in [4]), with most of them falling into one of the two distinct categories. The first (and by far the largest) group of methods attempts to predict mutation effects from amino acid sequences based on a broad spectrum of features, of which physicochemical properties of amino acids, evolutionary conservation, predicted structural properties, such as solvent accessibility, co-evolution with other residues, and functional annotation belong to the most frequently used ones. Integrated consensus classifiers as well as databases of pre-computed predictions are readily available [5]; [6]. The second group of methods assesses the consequences of residue substitutions based on three-dimensional structures of proteins, often in combination with sequence-derived attributes. The obvious disadvantage of this type of methods is that they are only applicable to the minority of proteins for which an experimental atomic structure or at least a high-quality homology model is available. On the up side, structure-based prediction methods are more accurate and potentially able to provide a mechanistic understanding of the damaging effect at the molecular level, which sequence-based methods generally cannot do. Structure-based methods typically analyze the influence of mutations on protein stability by predicting free energy changes (ΔΔG) using either energy functions or machine learning [7]. For example, CUPSAT [8] and I-Mutant2.0 [9] predict experimentally measured ΔΔG values at around 80% accuracy by employing coarse-grained atom and torsion angle potentials and a support vector machine, respectively. A recently published method, STRUM [10], assesses stability changes upon mutation and identifies disease-associated mutations based on predicted low-resolution protein models, i.e. when a 3D structure is actually not available. These and other methods do not explicitly predict the pathogenicity of mutations, although they are frequently used for this purpose based on the assumption that disease relevant mutations exert a more drastic effect on protein structure than benign mutations do and that changes of stability directly affect protein function. Although there is some correlation between disease-associated mutations and change in protein stability, many disease-related variants do not affect protein stability [11].

None of the methods discussed above explicitly take into account the folding arrangements of proteins and the overall structural context in which mutations occur. In particular, no specialized methods exist to predict mutation effects in transmembrane proteins, which constitute approximately 25–30% of proteins in each living cell [12] and a major proportion of all drug targets. Membrane proteins reside in the lipid environment and therefore evolve under markedly different structural constraints compared to globular proteins, with disease-causing mutations primarily occurring in buried positions of transmembrane helices [13]. According to the MutHTP database [14])over 170,000 disease-relevant mutations in transmembrane proteins have been accumulated in the public repositories of variation data, and 392 of them are associated with proteins of known 3D structure, which makes it possible to develop prediction models specific to transmembrane proteins. In this study we developed a machine-learning approach to discriminate between disease-associated and benign point mutations in transmembrane proteins with known 3D structure. The method, called BorodaTM (**BO**osted **R**egressi**O**n trees for **D**isease-**A**ssociated mutations in **T**rans**M**embrane proteins), was trained on sequence-, structure-, and energy-derived descriptors. When compared with the general purpose state-of-the art methods, BorodaTM is superior in classifying point mutations in transmembrane proteins. In a large-scale modelling study, we applied BorodaTM to classify each of the 19 possible amino acid substitutions in each sequence position for all human transmembrane proteins with a known 3D structure obtained from the PDBTM database. These predictions are available via the MutHTP database at https://www.iitm.ac.in/bioinfo/MutHTP/boroda.php.

## Methods

### Mutations in human transmembrane proteins with known topology

Human mutation data was obtained from the manually curated UniProtKB/Swiss-Prot variant database [15]. Benign polymorphisms were distinguished from disease-associated mutations based on the keywords “Polymorphism” and “Disease” in the “Type of variant” field. Variants annotated as “Unclassified” were ignored. Topology data for human transmembrane proteins with known three-dimensional structure was obtained from the Human Transmembrane Proteome (HTP) database [16]. Although a useful mapping of human variation on membrane protein sequences already exists [17], we chose to re-create such mapping independently in order to extract from the HTP database a number of required annotation attributes: Swiss-Prot accession number, sequence position of the mutation, amino acid change, specific evidence used to determine protein topology, location of the mutation with respect to the membrane (”M”-membrane, “L”-membrane reentrant loop, “I”-inside, “O”-outside, “S”-signal, and “T”-transit), as well as the identifiers of three-dimensional structures according to the Protein Data Bank [18]. Only point mutations located in the transmembrane regions of proteins were considered in this study. This yielded a preliminary dataset of 3888 point mutations, including 2309 disease-associated and 1579 benign mutations, associated with known structures. A non-redundant dataset reflecting variation in the transmembrane regions was created by excluding: i) those proteins that share more than 50% sequence identity, and ii) those proteins that share more than 75% structural similarity, as measured by the TMalign software [19]. This procedure yielded the final dataset of 392 disease-associated and 154 benign mutations in 64 proteins with the number of transmembrane alpha helices (TMs) ranging from 1 to 13 (see S1 Table).

Due to the stringent criteria we used to select these 64 proteins from more than 5000 membrane proteins contained in the human proteome (availability of known 3D structure, sequence redundancy, availability of mutation data, etc), but also due to the fact that proteins get chosen for crystallographic analysis based on subjective (medical interest) or objective (crystallization potential) criteria, this dataset would be expected to be biased in terms of its functional repertoire. Interestingly, however, we did not find any significant bias in terms of “Molecular Function”, as defined by the Gene Ontology [20], with the exception of “Transmembrane receptor activity” (see Supplementary file). Gene Ontology terms from the Cellular Components and Biological Processes subontologies enriched in our dataset are mostly related to cellular localization and some regulatory functions. Such biases can only be addressed in the future once more mutation and structural data become available.

### Descriptors of mutations

Each point mutation was represented by a vector in a multidimensional feature space, where each vector coordinate corresponds to a certain characteristic of the mutation. We will refer to such vectors as mutation descriptors. Our method incorporates sequence-, structure-, and energy-based descriptors initially used in the CompoMug method [21] to assess stability-enhancing effects of point mutations in G protein-coupled receptors. Recently, we also showed that leveraging such a broad spectrum of complementary features allows to derive more powerful prediction models [22].

#### Sequence-based characteristics of mutations

We used the AAindex database [23] as a source of physico-chemical properties of amino acids and selected a set of 12 characteristics: hydrophilicity (KUHL950101), amphiphilicity (MITS020101), bulkiness (ZIMJ680102), polarity (GRAR740102), polarizability (CHAM820101), isoelectric point (ZIMJ680104), accessible surface area in a tripeptide (CHOC760101), number of hydrogen bond donors (FAUJ880109), net charge (KLEP840101), radius of gyration of the side chain (LEVM760105), amino acid composition (CEDJ970103), and side-chain contribution to protein stability (TAKK010101). These properties were determined both for the wild-type and the mutant type of amino acid residues, resulting in 24 descriptor coordinates. Mutations were further characterized by four substitution scores: the Blosum62 substitution score, which is widely used in protein sequence analysis [24]; the PHAT score, which is analog to the Blosum62 score computed specifically for the membrane proteins [25]; and the two SLIM scores, which, in contrast to other substitution matrices, takes into account asymmetry in the observed mutations [26]. Altogether, 28 sequence-based descriptor components were derived.

#### Structure-based characteristics of mutations

Given the atomic structures of the wild-type proteins, we created structural models of the mutated proteins. The corresponding side chains were replaced and then all the neighboring side chains in the 5Å proximity were subjected to Monte-Carlo optimization in order to remove energetic stress caused by the point mutation. Main chain conformation was kept fixed. Subsequently we calculated for each mutation three sets of structure-based characteristics. The first set of characteristics reflects the secondary structure type of the mutated position and is encoded as a 6-bit categorical variable, with each bit representing α-helix, 3/10-helix, π-helix, β-strand, β-bridge, or coil. The second set of characteristics is related to the residue exposure to solvent. We computed the solvent accessible surface area (SASA) and the relative SASA, which is the ration of the actual and the maximum possible SASA for a given residue. A 3-bit categorical variable was defined, where each bit represents buried (relative SASA < 0.17), exposed (relative SASA > 0.43), or partially exposed (0.17 < relative SASA < 0.43) residues [22, 27]. The third set of characteristics is related to the contacts formed between the wild-type and mutated residues and the residues in their 5Å proximity. These include the total contact area, the total volume of the side chain, the number of hydrogen bonds, and the packing density, defined as the ratio of the molecular surface area and the solvent accessible surface area. We also recorded the number of polar, charged, aromatic, aliphatic, and special contacts formed by the mutated residue. Polar contacts comprise interactions with Asn, Gln, Thr, Ser, Tyr, and His residues, charged contacts—with Lys, Arg, Asp, and Glu residues, aliphatic contacts—with Ala, Leu, Ile, Val, and Met residues, aromatic contacts—with Phe, Trp, Tyr, and His residues, and special contacts—with Cys, Pro, and Gly residues. We used the ICM-Pro software (Molsoft L.L.C) to perform structure analysis and the CompoMug scripts [21] to compute structure-based characteristics. Altogether, 44 structure-based descriptor components for each mutation were computed.

#### Energy-based characteristics of mutations

For each mutated residue in the wild-type and mutant-type structural models we computed the electrostatic, Van-der-Waals, hydrogen-bond, entropic, and solvation energy, as well as the total energy as the sum of all individual energy terms. In addition, we calculated the total energies for all residues in the 5Å neighborhood from the target residue and used their sum normalized by the number of residues as an additional characteristic. Energy calculations were also conducted by ICM-Pro and CompoMug. Altogether, for the wild-type and mutant-type structural models we computed 24 energy-based components for the descriptor.

### Machine learning

The training matrix for machine learning was constructed by computing descriptors for each point mutation in the training dataset, and by labelling the descriptor with +1, if the corresponding point mutation is associated with a pathology, or with -1 otherwise. Identification of disease-associated mutations is thus cast as a binary classification problem, where the derived prediction model assigns labels +1 and -1 for a given point mutation to be disease-associated or benign, respectively. To train the prediction model we used the XGBoost software (http://xgboost.readthedocs.io/en/latest/), which provides a fast and powerful implementation of the gradient boosting decision tree algorithm [28]. We used a cross-validation procedure along with the t-statistic criterion to obtain the optimal number of estimators for the classifier, *i*.*e*. the number of decision trees in the model. Specifically, the entire dataset was randomly split into training (90%, 362 and 129 of disease-associated and benign mutations, respectively) and test (10%, 30 and 25 of disease-associated and benign mutations, respectively) data. Then a 5-fold cross validation was performed 20 times on the training set, and the t-statistic criterion was used to determine the optimal number of estimators for the prediction model. The test set was then used to estimate the performance of the prediction models obtained during the cross-validation step. The maximum number of features to consider when splitting a node in the tree was set to be equal to the square root of the descriptor size and no restraints were imposed on the depth of the tree. In addition, descriptors were normalized to have a zero mean and a unit variance. To cope with the imbalance in the training data we also weighted the descriptors by the factor of *N*_−1_/(*N*_−1_+*N*_+1_) and *N*_+1_/(*N*_−1_+*N*_+1_) for the disease-associated and benign mutations, respectively, where *N*_+1_ is the number of disease associated mutations, and *N*_−1_ is the number of benign mutations.

To evaluate the prediction power of the derived classifiers we calculated several standard characteristics:

- accuracy: $\frac{TP+TN}{TP+TN+FP+FN}$
- precision: $\frac{TP}{TP+FP}$
- recall: $\frac{TP}{TP+FN}$
- f-measure: $2\frac{Precision\times Recall}{Precision+Recall}$
- Matthews correlation coefficient (MCC): $\frac{TP\times TN-FP\times FN}{\sqrt{\left(TP+FP)\times (TP+FN)\times (TN+FP)\times (TN+FN\right)}}$

where TP (true positives) is the number of correctly classified disease-associated mutations, TN (true negatives) is the number of correctly classified benign mutations, FP (false positives) is the number of disease-associated mutations misclassified as benign, and FN (false negatives) is the number of benign mutations misclassified as disease-associated.

To evaluate the feature importance in the obtained prediction models we calculated a feature gain (FG) by averaging gains on each branch of the tree, which are defined as:

$$FG=\frac{1}{2}(\frac{f_{\alpha }(G_{jmL}{)}^{2}}{H_{jmL}+\lambda }+\frac{f_{\alpha }(G_{jmR}{)}^{2}}{H_{jmR}+\lambda }-\frac{f_{\alpha }(G_{jm}{)}^{2}}{H_{jm}+\lambda })-\gamma$$

where *f*_*α*_(*x*) = {*x*+*α*, *if x*<−*α*;*x*−*α*, *if x*>*α*; 0, *otherwise*}; *γ*, *λ*, and *α* are the penalization, L2 regularization, and L1 regularization terms, respectively; *j* and *m* stand for the current branch and estimate, respectively; *G* and *H* are the gradient and the Hessian of the loss function. All calculations were performed with the scikit-learn python modules [29].

## Results

### Prediction model

**Fig 1:**
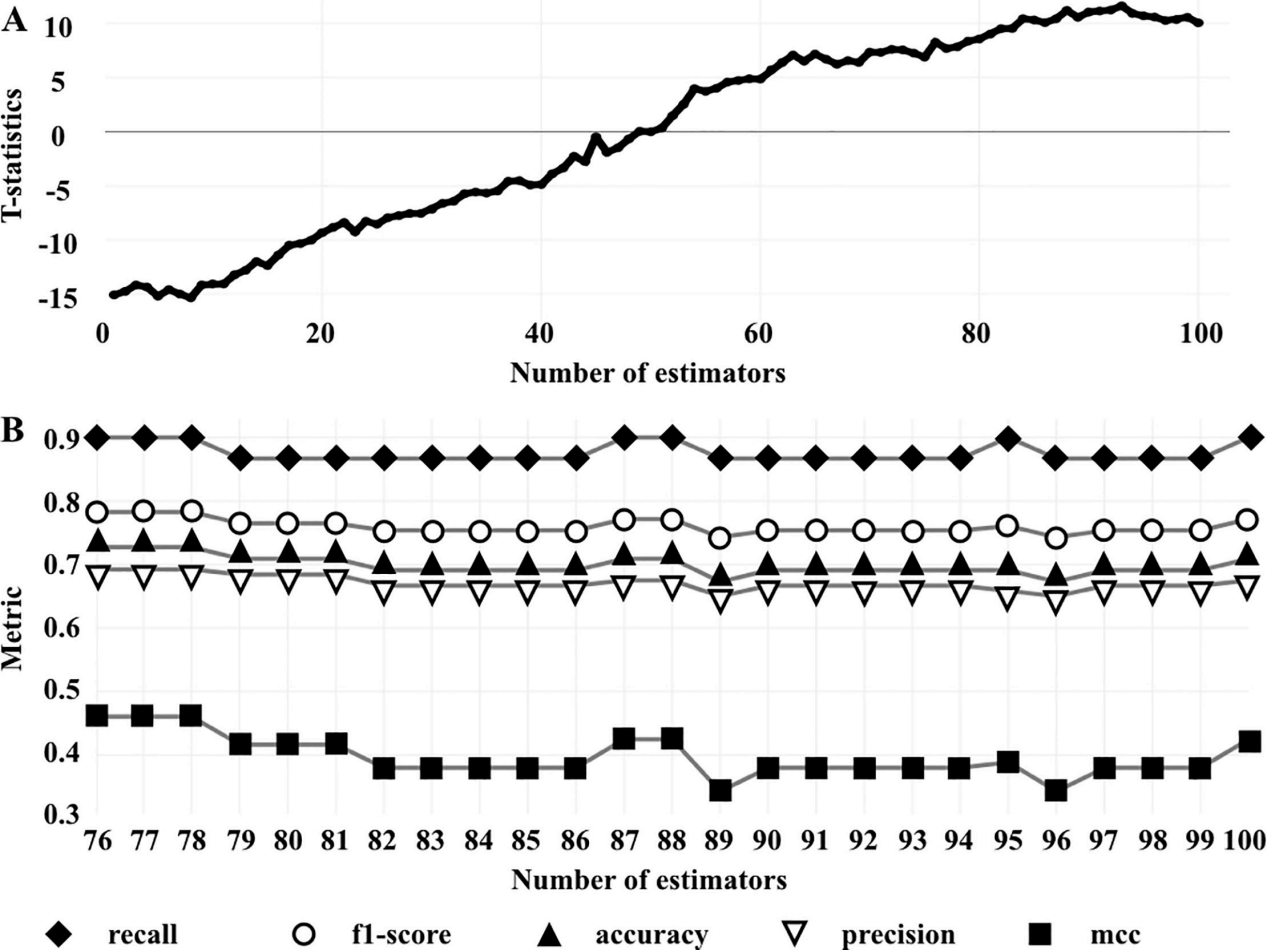
Evaluation of the derived prediction models. A) T-statistics calculated for the derived prediction models with 1, 2, …, 100 estimators w.r.t. the prediction model with 50 estimators. B) Performance metrics on the test set for the best 25 prediction models in terms of t-statistics.

Our dataset consists of 392 disease-associated and 154 benign point mutations located in 64 transmembrane proteins. For each point mutation in our dataset we computed descriptors, generated the training and test datasets, and determined the optimal number of estimators using the t-statistic criterion, as described in the Methods section. The number of estimators was set to the values between 1 and 100 to derive 100 prediction models. The performance of each model was assessed relative to the prediction model with 50 estimators (Fig 1A). As expected, the prediction power grows with the number of used estimators, achieving the best performance with 93 estimators in terms of t-statistics. Then we evaluated the performance of the best 25 prediction models on the test set and identified the optimal number of estimators in terms of the performance characteristics (see the Methods section). As seen in Fig 1B, models corresponding to 76, 77, and 78 estimators have the highest characteristics on the test set, as compared to the other models. We selected 77 as the optimal number of estimators. The performance metrics of the prediction model with 77 estimators for the train, test, and entire datasets are presented in Table 1. The model performs well on the training set, implying that the constructed features capture relevant information to describe disease-associated point mutations. Furthermore, the obtained model also performs well on the test set, implying that there is no significant overfitting to the training data. However, we would like to note that due to the imbalance in the training set (392 disease-associated vs 154 benign mutations), there might be a bias towards prediction of disease-associated mutations, compared to the benign mutations. Indeed, precision is very high on the training set (99.7%) but significantly lower on the test set (69.2%). We believe that such deficiencies are related to the relatively small dataset and that accumulation of relevant structural data will result in better prediction models.

**Table 1:** Benchmark results of the prediction model.

Metric	Training set	Test set
Accuracy	94.3 %	72.7 %
Precision	99.7 %	69.2 %
Recall	92.5 %	90.0 %
F1	96.0 %	78.3 %
MCC	0.87	0.46
Number of true positives	335 (68.2%)	27 (49.1%)
Number of true negatives	128 (26.1%)	13 (23.6%)
Number of false positives	1 (0.2%)	12 (21.8%)
Number of false negatives	27 (5.5%)	3 (5.5%)

### Feature selection

We next attempted to identify the most informative sequence-based, structure-based, and energy-based features using the optimal prediction model with 77 estimators based on the feature gain calculated for each feature. Features with *FG*<0.01*FG*_*max*_ (the maximum FG over all features) were filtered out, resulting in 41 most informative features (S2 Table). While all three types of features (10 sequence-based, 15 structure-based, and 16 energy-based) proved to be important, more structure- and energy-based features got selected, indicating that they are informative for the classification task. To verify that there is no information loss due to feature selection, we derived a prediction model with a reduced set of selected features and an optimal number of estimators and achieved the same performance on the training and the test sets. In contrast, using of single type of features, e.g. solely sequence-, or structure-, or energy-based descriptors, resulted in significantly lower MCC values on the test set (0.30, 0.15, and 0.22 for the sequence-, structure-, and energy-based descriptors, respectively), as compared to the combined feature set (0.46). Finally, we re-derived a prediction model using the obtained optimal parameters and the entire dataset. We will refer to this prediction model as BorodaTM, which stands for **BO**osted **R**egressi**O**n trees for **D**isease-**A**ssociated mutations in **T**rans**M**embrane proteins.

### Predictions are not biased towards specific mutation types or proteins

**Fig 2:**
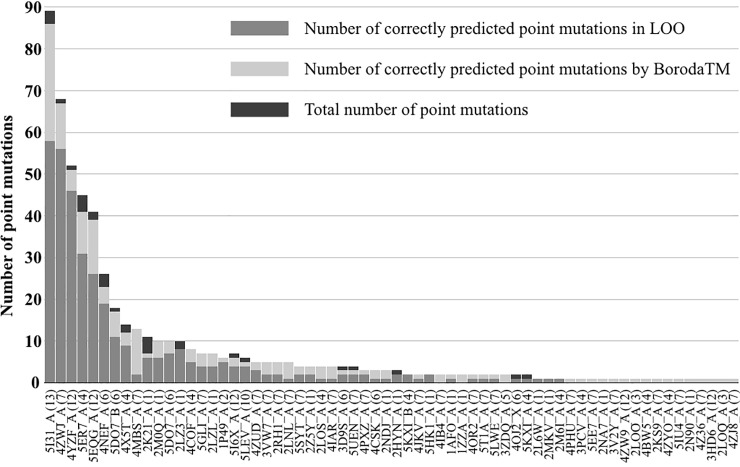
Distribution of point mutations across proteins. Black and light gray bars correspond to the total number of point mutations and the number of point mutations correctly predicted by the optimal prediction model, respectively. Dark grey bars correspond to the number of correctly predicted point mutations in the leave-one-out validation (LOO).

The dataset comprises 546 point mutations located in 64 proteins of different topology (see S1 Table). Since mutations are very unevenly distributed over individual proteins, we sought to verify that the optimal prediction model is not biased towards any specific proteins by measuring the number of correctly classified mutations for each protein separately. As it seen in Fig 2 (light grey bars), the obtained prediction model is sufficiently robust and correctly classifies at least one point mutation in each protein.

There are 380 possible amino acid substitutions, with some of them being much more likely than the others. S3 Table shows the frequency of amino acid substitutions actually observed in our dataset. Some mutations (e.g. R->C/Q/H/W) are strongly over-represented, while 269 mutations (68% of all possible mutations) are completely missing. The absence of certain amino acid substitutions limits the prediction power of the derived classifiers, but it is hoped that this limitation will become less severe as more data become available. We evaluated the ratio of correctly classified exchanges between all pairs of amino acids and found that the performance of our prediction model remains relatively stable for all point mutation types and is not biased towards any particular residue pair (see S3 Table).

### Leave-one-out validation

Ideally, prediction models should be evaluated on a completely independent dataset with no relation to the data used to develop the method. However, an independent dataset of disease-associated and benign point mutations completely unrelated to the human variation data used in this study does not appear to exist. We therefore performed leave-one-out cross-validation on a dataset covering non-redundant point mutations in 64 different proteins. We derived 63 prediction models, each trained on one of the 63 possible subsets, and evaluated the performance of these models with respect to the point mutations in the sole excluded protein. As seen in Fig 2 (grey bars), the prediction model is able to correctly classify point mutations in proteins not contained in the training dataset. For example, for three proteins of different topology with the largest number of point mutations (Nieman-Pick C1 protein, 13TMs; rhodopsin, 7TMs; and anion exchanger, 12TMs), the corresponding prediction models correctly classified 61 out of 89, 57 out of 68, and 47 out of 52 point mutations, respectively. The ratio of correctly predicted mutations in this test was 65.4%, which is comparable with the accuracy achieved on the test set (72.7%). However, there are a number of cases (20), when a protein is represented by at most three point mutations, which were not correctly classified by the prediction models (see Fig 2).

### Comparison with the state-of-the-art predictors

To the best of our knowledge, BorodaTM is the first predictor designed to classify disease-associated point mutations specifically in transmembrane regions. We therefore compared it with Entprise[30], the currently leading general-purpose prediction method based on the boosted tree regression approach. Entprise uses sequence- and structure-level information for discriminating between the disease-associated and benign point mutations, where upon 3D information is obtained by structure prediction. It was reported to outperform other state-of-the-art prediction tools, such as PolyPhen-2 [1], FathHmm [31], Sift [32], MutationAssesor [33], and MutationTaster [34]. Entprise was trained on a previously constructed dataset [35], which overlaps with the HumVar dataset. The Entprise web-server (http://cssb2.biology.gatech.edu/ENTPRISE/) provides a pathology score for each point mutation in its training set, with pathology scores > 0.45 assumed to correspond to disease-associated mutations. Given that there is a crystallographic structure for each point mutation in our benchmark and that Entprise and our method use this information for training, it is fair to compare our method with Entprise. To this end, we retrieved pathology scores for 386 disease-associated and 151 benign point mutations present both in our and Entprise’s training sets and evaluated the performance of the two methods. As seen in Table 2, our prediction model significantly outperforms Entprise in terms of accuracy, precision, f1 (~8% better performance for each metric), and the mcc (0.22 better performance) metric, and slightly outperforms it in terms of the recall metric (1.7% better performance).

**Table 2:** Comparison of BorodaTM and Enterprise predictions for point mutations in the transmembrane and soluble regions.

	Transmembrane regions				Soluble regions			
Metric	BorodaTM	Entprise	Polyphen-2	SIFT	BorodaTM	Entprise	Polyphen-2	SIFT
Accuracy	**93.5 %**	85.5 %	80.0 %	79.3 %	62.5 %	**83.0 %**	75.7 %	73.8 %
Precision	**99.2 %**	91.7 %	83.2 %	82.3 %	64.5 %	**89.0 %**	73.1 %	71.1 %
Recall	**91.7 %**	90.0 %	90.2 %	90.5 %	71.9 %	83.1 %	**86.3 %**	85.2 %
F1	**95.3 %**	88.3 %	86.6 %	86.2 %	68.0 %	**86.0 %**	79.2 %	77.5 %
MCC	**0.86**	0.64	0.47	0.45	0.23	**0.65**	0.51	0.47

### Performance of BorodaTM on point mutations in soluble regions

To further assess the prediction power of BorodaTM, we investigated how well our prediction model, trained on point mutations in transmembrane regions, discriminates point mutations in soluble domains of transmembrane proteins, which exhibit vastly different physicochemical properties. We collected a dataset of 2882 point mutations, including 1597 disease-associated and 1285 benign mutations, located in the solvent-accessible extra- and intra-cellular domains of transmembrane proteins. For comparison, we retrieved predictions for these mutations from the Entprise, Polyphen-2, and SIFT methods. As expected BorodaTM exhibited a much lower success rate (Table 2), as compared to that of the other methods. Therefore, our method it its present form is not well suited for handling extramembranous portions of proteins. Nonetheless, the method correctly classified ~2/3 (62.5%) of the point mutations in soluble regions, indicating that the model can be adapted to the classification of point mutations located in the soluble regions, if re-trained on the corresponding dataset.

### Availability

We applied BorodaTM to conduct a large-scale mutation analysis of the 379 human membrane proteins with known 3D structures available from the PDBTM database (version 06–2017) [36]. For each protein we generated structural models for all point mutations by replacing each residue to 19 possible residue types. Each structural model was refined and prepared for the descriptor computation, similarly to the training set. This procedure was followed by the computation of the sequence-based, structure-based and energy-based descriptors, resulting in ~1.8 million feature vectors. Note that we could not compute descriptors for poorly resolved or missing residues in the PDB structure. Finally, we applied BorodaTM to classify each point mutation as benign or diseased associated. In addition, we classified each point mutation using pre-computed results of the Sift [32], Provean [37], and Polyphen-2 [38] web-servers. All predictions are available at https://www.iitm.ac.in/bioinfo/MutHTP/boroda.php, where users can search the data using either PDB or Uniprot ID and download the entire dataset. The ICM scripts for the descriptor computations can be found at https://gitlab.com/pp_lab/CompoMug, and the derived prediction model along with the training dataset can be found at https://gitlab.com/pp_lab/boroda.

## Conclusions

In this study we developed BorodaTM—a machine learning tool, which discriminates between disease-associated and benign point mutations in transmembrane regions. BorodaTM uses sequence-based, structure-based, and energy-based information derived from wild-type and mutant-type amino-acid residues and their 5Å neighborhood. BorodaTM was trained on a non-redundant set of 546 point mutations located in 64 proteins of different topology, resulting in 92.5% and 90.0% recall values on the training and test sets, respectively. It significantly outperforms the state-of-the-art approach, Entprise, which was trained on a larger dataset, including point mutations in both soluble and membrane proteins. Surprisingly, BorodaTM also performs relatively well for the point mutations located in soluble regions of the transmembrane proteins. We applied BorodaTM to classify ~1.8 millions point mutations in the transmembrane regions of 379 human proteins with known 3D structure. These results are provided at https://www.iitm.ac.in/bioinfo/MutHTP/boroda.php together with predictions made by a number of other publicly available methods.

## Supporting information

S1 Fig A dotplot showing significantly overrepresented GO terms for the transmembrane proteins with 3D structures. (DOCX)

S1 Table Training dataset. (XLSX)

S2 Table List of features. (XLSX)

S3 Table Distribution of amino acid substitutions in the training dataset. (DOCX)
