# Supplementary material for: Prediction of disease-associated mutations in the transmembrane regions of proteins with known 3D structure
Source: PLoS One. 2019 Jul 10;14(7):e0219452. doi: 10.1371/journal.pone.0219452 (PMC6620012; doi:10.1371/journal.pone.0219452)
Supplement: S1 Fig — (DOCX) [file pone.0219452.s001.docx]

To find significantly overrepresented GO terms for the proteins in our dataset, we used DAVID^1^. For a GO term to be considered significantly overrepresented, we set a Bonferroni adjusted p-value cut off of < 0.05. Visualization of the GO terms as dotplots was carried out in R.

S1 Fig. A dotplot showing significantly overrepresented GO terms (MF - Molecular Function, CC- Cellular Component and BP - Biological Processes) for the transmembrane proteins with 3D structures. The dot sizes indicate the number of proteins within a GO term, the bigger the size of the dot the more the proteins in that ontology.

**References**

1. Huang, D. W. *et al.* DAVID Bioinformatics Resources: expanded annotation database and novel algorithms to better extract biology from large gene lists. *Nucleic Acids Res.* **35**, W169-175 (2007).
