# Supplementary material for: Prediction of disease-associated mutations in the transmembrane regions of proteins with known 3D structure
Source: PLoS One. 2019 Jul 10;14(7):e0219452. doi: 10.1371/journal.pone.0219452 (PMC6620012; doi:10.1371/journal.pone.0219452)
Supplement: S3 Table — (DOCX) [file pone.0219452.s004.docx]

**Table S3.**Distribution of various amino acid substitutions in the training dataset. Each cell is of type *a/b*, where a and b are the number of correctly classified point mutations and the total number of point mutations, respectively.

|  | **A** | **R** | **N** | **D** | **C** | **E** | **Q** | **G** | **H** | **I** | **L** | **K** | **M** | **F** | **P** | **S** | **T** | **W** | **Y** | **V** |
| --- | --- | --- | --- | --- | --- | --- | --- | --- | --- | --- | --- | --- | --- | --- | --- | --- | --- | --- | --- | --- |
| **A** | - | - | - | 3/3 | - | 5/5 | - | 1/1 | - | - | - | - | - | - | 3/3 | 3/4 | 8/9 | - | - | 12/14 |
| **R** | - | - | - | - | 16/17 | - | 13/15 | 3/3 | 23/24 | - | 7/7 | - | - | - | 4/4 | 5/5 | - | 19/19 | - | - |
| **N** | - | - | - | 0/1 | - | - | - | - | - | 4/4 | - | 3/4 | - | - | - | 9/9 | 1/1 | - | 1/1 | - |
| **D** | 1/1 | - | 12/13 | - | - | 1/1 | - | 1/1 | 3/3 | - | - | - | - | - | - | - | - | - | 5/5 | 2/2 |
| **C** | - | 4/4 | - | - | - | - | - | - | - | - | - | - | - | 2/2 | - | 1/1 | - | 3/3 | 2/2 | - |
| **E** | - | - | - | 2/2 | - | - | 2/3 | 1/1 | - | - | - | 11/11 | - | - | - | - | - | - | - | 1/1 |
| **Q** | - | - | - | - | - | 3/3 | - | - | 1/2 | - | 1/1 | 3/3 | - | - | 4/4 | - | - | - | - | - |
| **G** | 3/3 | 18/19 | - | 9/9 | 2/2 | 5/5 | - | - | - | - | - | - | - | - | - | 11/12 | - | 1/1 | - | 5/5 |
| **H** | - | 3/3 | - | 1/1 | - | - | 1/1 | - | - | - | - | - | - | - | 2/2 | - | - | - | - | - |
| **I** | - | - | 2/2 | - | - | - | - | - | - | - | 2/2 | 1/1 | 1/1 | 4/4 | - | 1/1 | 4/4 | - | - | 7/8 |
| **L** | - | 4/4 | - | - | - | - | 2/2 | - | 1/1 | - | - | - | 1/1 | 3/3 | 13/14 | - | - | - | - | 4/5 |
| **K** | - | 1/1 | 3/3 | - | - | 3/3 | 1/1 | - | - | - | - | - | - | - | - | - | - | - | - | - |
| **M** | - | 3/3 | - | - | - | - | - | - | - | 2/3 | 3/3 | 3/3 | - | - | - | - | 4/4 | - | - | 4/4 |
| **F** | - | - | - | - | 3/3 | - | - | - | - | - | 12/14 | - | - | - | - | 1/1 | - | - | - | 2/2 |
| **P** | 2/2 | 4/4 | - | - | - | - | 3/3 | - | 1/1 | - | 11/11 | - | - | - | - | 5/5 | 2/3 | - | - | - |
| **S** | - | 2/2 | 1/1 | - | 2/2 | - | - | 2/2 | - | 3/3 | 4/8 | - | - | 4/4 | 6/7 | - | 1/1 | - | - | - |
| **T** | 3/5 | 4/4 | - | - | - | - | - | - | - | 7/7 | - | 4/4 | 9/10 | - | - | 1/1 | - | - | - | - |
| **W** | - | 4/4 | - | - | 4/4 | - | - | - | - | - | - | - | - | - | - | 2/2 | - | - | - | - |
| **Y** | - | - | 2/2 | - | 7/7 | - | - | - | 2/2 | - | - | - | - | 2/2 | - | - | - | - | - | - |
| **V** | 4/4 | - | - | 1/1 | - | 1/1 | - | 3/3 | - | 9/12 | 5/6 | - | 16/17 | 3/3 | - | - | - | - | - | - |
